# Impairment of Spatial Working Memory but Preservation of Recognition Memory in Female Rats with Spontaneous Absence Seizures

**DOI:** 10.1007/s11064-025-04485-w

**Published:** 2025-07-17

**Authors:** Mariana Neuparth-Sottomayor, Tatiana P. Morais, Mark Good, Ana Maria Sebastião, Giuseppe Di Giovanni, Vincenzo Crunelli, Sandra H. Vaz

**Affiliations:** 1 Instituto de Farmacologia e Neurociências, Faculdade de Medicina, Universidade de Lisboa, Lisboa, Portugal; 2 CCUL (CCUL@RISE), Faculdade de Medicina, Centro Cardiovascular da Universidade de Lisboa, Universidade de Lisboa, Lisboa, Portugal; 3 Neuroscience Division, School of Bioscience, Cardiff University, Cardiff, UK; 4 School of Psychology, Cardiff University, Cardiff, UK; 5 Department of Medical and Surgical Sciences, University of Magna Graecia, Catanzaro, Italy; 6 Gulbenkian Institute for Molecular Medicine (GIMM), Lisbon, Portugal

**Keywords:** Comorbidities, Anxiety, GAERS rats, NEC rats, Wistar rats, NOR, EPM, Y maze

## Abstract

Epidemiological studies reveal gender-specific differences in epilepsy. Childhood absence epilepsy (CAE), which is more prevalent in females, is characterized by typical absence seizures (ASs) consisting of brief periods of unconsciousness, associated with 2.5–4 Hz spike-wave discharges (SWDs) in the electroencephalogram (EEG). Children with CAE often present neuropsychological comorbidities, including deficits in attention and executive function. In this study, we investigated anxiety-like behaviour and memory in female Genetic Absence Epilepsy Rat from Strasbourg (GAERS), a validated model of ASs, compared to Non-Epileptic Control (NEC) and Wistar rats. We found that female GAERS generally showed normal anxiety-like behaviour relative to both control strains, although some tests suggested a reduction in anxiety. Importantly, female GAERS showed impaired spatial working memory, while recognition memory was preserved. These findings when compared with previous data in males indicate that while anxiety levels in female GAERS are preserved as those of male GAERS, memory performance differs, with males showing impairments in both spatial working memory and recognition memory. These findings emphasize the importance of considering gender differences in both clinical and preclinical epilepsy research to better understand the neuropsychological comorbidities associates with ASs. This knowledge is crucial for the identification of gender-specific mechanism, as well as the development of gender-sensitive, personalized therapies targeting both seizures and associated cognitive impairments.

## Introduction

Epidemiological studies have revealed gender-specific differences in epilepsy expression and prevalence, as well as in their comorbidities. Although the global prevalence and rates of unprovoked seizures, status epilepticus, and tonic-clonic seizures are higher in males [1–3], some epilepsies, such as juvenile myoclonic epilepsy and childhood absence epilepsy (CAE), are more prevalent in females [1, 2, 4].

A common trait of epilepsy comorbidities includes anxiety, which tends to be more frequent in females than in males (35.5% in females versus 23.2% in males) [5–7]). In males with CAE, anxiety is associated with younger age, seizure frequency and psychosocial factors, including sleep quality, whereas in females it mostly depends on seizure frequency only [5, 6]. Cognitive impairments are also prevalent among people with epilepsy, affecting memory, attention and executive function. These are mainly related to epilepsy duration, age of onset, seizure frequency, and anti-seizure medication (ASMs) [8].

CAE, the most prevalent epilepsy in children, is solely characterized by the presence of absence seizures (ASs), that are sudden impairments of consciousness, coupled with bilaterally synchronous 2.5–4 Hz spike-and-wave discharges (SWDs) on the electroencephalogram (EEG) [9, 10]. Neuropsychological comorbidities, including deficits in attention, executive function, and memory [11, 12], are present in ~60% of children with CAE, and can persist into adulthood, even after seizure remission [13, 14]. To the best of our knowledge, no studies have investigated gender differences in anxiety and neuropsychological comorbidities in children with CAE. In general, females perform better on executive function tests, delayed face recognition memory and verbal learning tasks than males [8, 15–17] whereas in mesial temporal lobe epilepsy, males perform better than female on verbal intelligence quotient (IQ), performance IQ, and naming tasks [18]. This suggests that sex differences may also be present in CAE.

To characterize the pattern of deficits in female CAE, we used the well-validated rat model of ASs, the Genetic Absence Epilepsy Rat from Strasbourg (GAERS) that display 100% seizure prevalence regardless of sex [19, 20]. Most studies on experimental ASs do not report sex differences [21–23]. Early studies on cognitive impairments focused on male GAERS rats [24–26]. Notably, female GAERS do not consistently have stronger cognitive impairments than males; rather, the nature and severity of those deficits vary by cognitive domain. Males GAERS show greater impairments in visual discrimination and reversal learning, indicating difficulties with cognitive flexibility and adaptation [27]. In contrast, females exhibit more pronounced deficits in sociability, with reduced social approach behaviours compared to males [23]. These sex-specific patterns highlight the importance of considering sex as a biological variable in epilepsy research [22, 28]. When comparing published data from males and females that were performed in different labs using different colonies, it is difficult to draw conclusions about sex-differences. Indeed, GAERS colonies maintained in different establishments demonstrate phenotypic heterogeneity. Anxiety/depressive-like behaviour and cognitive impairment have been consistently reported in both sexes of GAERS rats form the Melbourne and Canadian colonies [22, 23, 27–30]. In contrast, data from the Strasbourg [25], Cardiff [31] and Maltese [32–34] colonies have shown inconsistent or low levels of anxiety-like behaviour in male GAERS compared to NEC rats. We recently showed that male GAERS from the Cardiff colony exhibit extensive cognitive impairment [31] that do not depend on high levels of anxiety-like behaviour. Moreover, anxiety testing in other absence epilepsy models (GAT1 knockout mice) has primarily focused on males, although two studies in Stargazer mice and WAG-Rij rats, found similarly low levels of anxiety-like behaviour in both males and females [35–38].

In the present study, we used three anxiety tasks and two memory tests in female GAERS rats and compared their results with the female inbred Non-Epileptic Control (NEC) rat strain from the Cardiff colony and the female outbred Wistar rats. We found that female GAERS displayed reduced neophobia in emergence test, while the level of anxiety-like behaviour is comparable to Wistar rats. Spatial working memory deficits were present in female GAERS rats, but object recognition memory was similar to that of the two control strains. Notably, females have low levels of anxiety similar to male GAERS rats from the same colony [31] but they differ in memory performance, with female GAERS rats showing deficits in spatial working memory but not in recognition memory, in contrast to male GAERS rats that show deficits in both tasks [31].

## Materials & Methods

### Animal Subjects

Adult (3–6-month-old) female GAERS and NEC rats from the Cardiff (UK) colony and female Wistar rats purchased from Charles River Laboratories (Lyon, France) were housed in groups of 3–4 animals in transparent plastic cages that contained cardboard tubes, nests, aspen woodblocks and bedding material. Animals were kept on a 12-hour light-dark cycle (light on at 7.00am) under constant temperature (22ºC) and humidity (80%) and were given *ad libitum* access to water and food. Animal age was equally distributed in the three strains in all tests and all animals completed the test battery in a daily counterbalanced order. All experiments were conducted during the light phase of the light-dark cycle and in conformity with European Community Guidelines (Directive 2010/63/UE) and the UK Animal Scientific Act, and under general guidance for animal epilepsy experimentation (Lidster et al., 2016). Care was taken in minimizing the number and suffering of the animals.

**Fig. 1:**
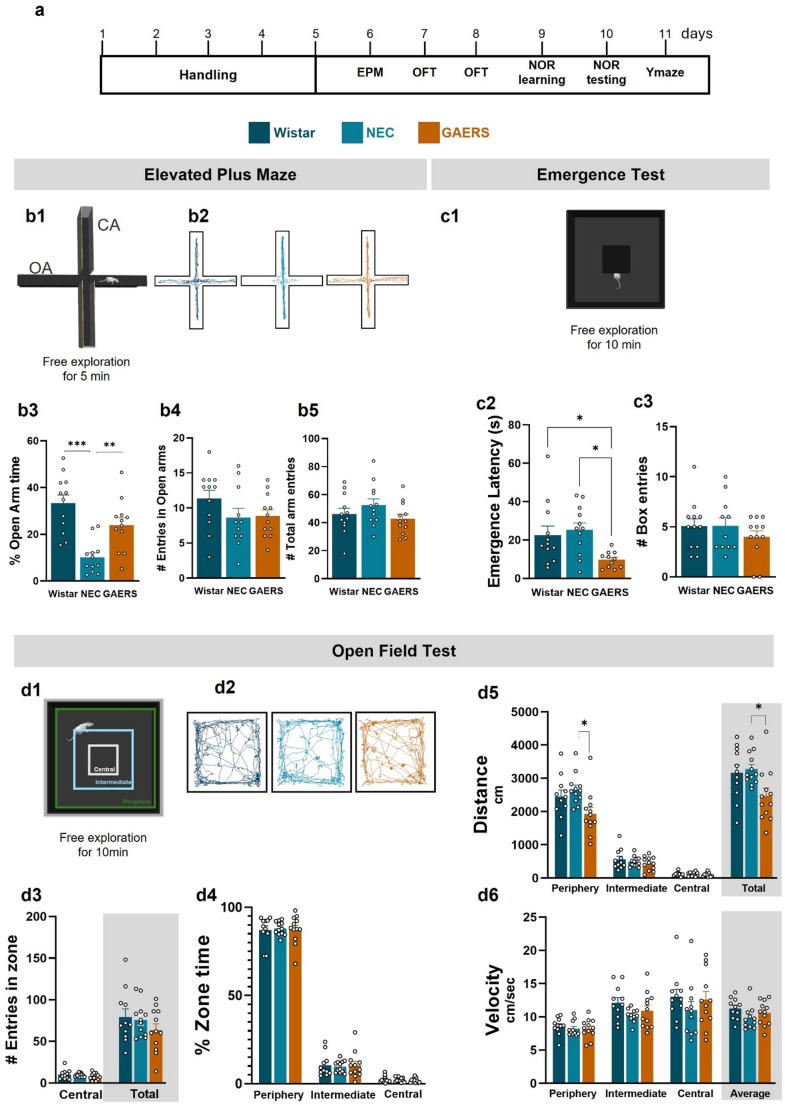
Anxiety-like behaviour in female GAERS. **a**) Timeline protocol of this study. **b1**) Representation of the elevated plus maze (EPM). **b2**) Illustrative traces of the activity of a Wistar, a NEC and a GAERS rat during 5 min free exploration of the EPM. **b3**) Time spent in open arms (OA) as a percentage of total testing time. **b4**) Number of entries in OA. b5) Number of total entries. **c1**) Representation drawing of the emergence test (ET) arena. **c2**) Latency of emergence from the central box. **c3**) Number of entries in the central box. **d1**) Representation drawing of the open arena with the indicated virtual central, intermediate and peripheral zones that was used for the open field test (OFT). **d2**) Illustrative traces of the activity of a Wistar, a NEC and a GAERS rat during the 10 min free exploration of the arena. **d3**) Number of entries in different zones. **d4**) Time spent in each zone. **d5**) Distance covered in each zone and total distance covered. **d6**) Velocity in each zone and average velocity. (Wistar *n* = 12, NEC *n* = 13, GAERS *n* = 12) (*p < 0.05, **p < 0.01, ***p < 0.001, one-way ANOVA followed by Tukey pairwise multiple comparisons tests)

All animals were acclimatized to the laboratory conditions for at least a week before the beginning of the experiments. Animals were then handled for 5 consecutive days (5 min/day) before the testing began (Fig. 1A). In each behavioural task, the testing order was randomized as was the rats starting position and the location of the object (where applicable). On the day of the experiments, the animals were placed for 1 h in the testing room for habituation and all equipment was cleaned with 30% ethanol between trials to remove olfactory cues. All trials were video-recorded using CamStudio and OBS studio^®^ (V. 27.1.3, 480 × 640) and the video-tracking software, SMART PANLAB^®^, (V3.0.06, Panlab, Harvard Apparatus, Barcelona, Spain). The reference point to determine the position of the animal was the center of the rat dorsum or head. Off-line analysis of tracking was performed using Solomon Coder (V. beta 19.08.02).

### Behavioural Tests

#### Elevated Plus Maze

The Elevated Plus Maze (EPM) was elevated 70 cm from the ground and consisted of two open arms (OA) crossing in the middle with two enclosed arms (CA) (Fig. 1b1) [39]. Animals were placed in the centre of the maze facing an open arm and allowed to explore the maze for 5 min. Mild illumination was used (42 lx). The total time spent in OA and the total number of arm entries were measured. Twelve Wistar, 13 NEC and 12 GAERS were used in this test. The primary outcome was the time spent in OA.

#### Emergence Test

The apparatus of the Emergence Test (ET) consisted of a 60 × 60 × 60 cm floor arena with high black acrylic walls and a square box (20 × 20 cm) in the middle with a cover and one lateral escape hole (Fig. 1c1). Mild illumination (42 lx) was used. Rats were placed in the box and the box was then placed in the middle of the open field. The box was then closed and the time required to emerge completely from the box as well as the number of entries in the box were recorded. Each animal performed a single trial of 10 min. Twelve Wistar, 13 NEC and 12 GAERS were used in this test. The primary outcome was the latency to exit the box.

#### Open Field Test

The open field (OF) consisted of 65 × 65 × 60 cm arena with high black acrylic walls and the trials were 10 min long (Fig. 1d1). Mild illumination was used (42 lx). The arena was virtually divided into three different square zones: a peripheral zone, an intermediate zone, and a central zone. Each rat was placed in the center of the arena facing north, east, south or west in a randomly assigned manner. The percentage of time in the central zone, as well as entries in the central zone were measured, and the number of faecal boli counted. In addition, thigmotaxis, total distance covered, and average velocity were analyzed as measures of locomotor activity. Twelve Wistar, 13 NEC and 12 GAERS used in this test. The primary outcome was the permanence in the central zone.

#### Novel Object Recognition

The novel object recognition (NOR) test was conducted in an open field arena (65 × 65 cm) (Fig. 3a) The rats were habituated to the apparatus for 3 days, 10 min/day in the absence of any objects. Subsequently, all rats received an object sample trial (on day 9), and a novel/familiar object test trial (on day 10) (Fig. 1a). The familiar or novel objects were randomized and the position relative to the other object was transposed on day 9. In the sample trial, the rat was placed in the arena with two familiar objects (*Pedras Salgadas*^®^ water bottle and a *Cristal*^®^ beer bottle) and allowed to freely explore the objects for 5 min. In the test trial (on day 10), the rats were allowed to freely explore the open field containing a familiar object and a novel object. Exploratory behaviour was quantified as the time touching or focusing an object (nose pointing in the direction of the object within a perimeter of 2 cm). The difference between time spent exploring novel and familiar objects in the total exploration time was defined as the novelty index (NI), and calculated as:

$$\begin{array}{l}\:{\rm{Novelty}}\:{\rm{Index}}\: = \\\frac{{{\rm{Time}}\:{\rm{exploring}}\:{\rm{the}}\:{\rm{novel}}\:{\rm{object}}\: - \:\:{\rm{Time}}\:{\rm{exploring}}\:{\rm{the}}\:{\rm{familiar}}\:{\rm{object}}}}{{{\rm{Total}}\:{\rm{time}}\:{\rm{exploration}}\:\left( {\rm{s}} \right)}}\end{array}$$

Trials were rejected if the animal jumped on the object or explored each object for less than 10 s in the sample phase. The total time exploring each object and the frequency of interactions with the objects, as well as the percentage of novel object exploration were recorded. Twelve Wistar, 13 NEC and 12 GAERS were used.

#### Y-Maze

Honig (1978) [40] defined working memory as information retained on any single trial that is required for performance only for that trial [41]. To test short-term spatial working memory, a Y-Maze composed of three symmetric arms (A, B and C), with 120º between arms, was used (Fig. 3a). The rat was placed at the end of a randomly assigned arm and allowed 8 min of free exploration of the apparatus. The total number of arm entries was recorded and the percentage of correct arm alternations (i.e., the animal visited a different arm than the one it arrived from) was quantified using the formula:

$$\begin{array}{l}\% \:{\rm{correct}}\:{\rm{alternations}}\:{\rm{ = }}\\\:\frac{{{\rm{Number}}\:{\rm{of}}\:{\rm{correct}}\:{\rm{alternations}}\:}}{{{\rm{Total}}\:{\rm{entries}}\: - \:2}} \times \:100\end{array}$$

Twelve Wistar, 13 NEC and 12 GAERS were used in this test. The primary outcome was the percentage of correct alternations.

### Statistics

Statistical significance was evaluated using GraphPad Prism (V. 9) for Windows^®^ and Rstudio^®^ (Version 2021.09.0 + 351). Data are reported in the text and figures as mean ± SEM of n independent observations in each experimental group. In behaviour analysis, each rat’s performance corresponds to an *n* value. The ROUT method was used for outlier identification. Unpaired two-tailed Student’s t-test was used for independent samples, to perform two-sample comparisons. One-way ANOVA followed by Tukey’s post-hoc test was used for multiple comparisons between more than two groups. Two-way ANOVA followed by Tukey’s post-hoc test was used to detect interactions between strain and day (independent variables) on the dependent variables.

## Results

### Anxiety-like Behaviour in the EPM

In the elevated plus maze (EPM) (Fig. 1b1), one-way ANOVA revealed a significant strain-dependent difference in the time spent in the OA (F_2,33_ =15.23; *p* < 0.001) (Fig. 1b3) (see representative traces in Fig. 1b2). Post-hoc analysis showed that GAERS spent 23.93 ± 3.2% of time in OA, which was similar to that of Wistar rats (33.30 ± 3.54%; *p* = 0.082) and significantly higher than NEC (10.1 ± 2%; *p* < 0.007). Nevertheless, no significant differences were found among strains in the number of OA entries (F_2,32_=1.77; *p* = 0.186) (Fig. 1b4), or in the total number of arm entries (F_2,33_=1.61; *p* = 0.22).

In summary, in the EPM test, female GAERS rats display anxiety-like behavior comparable to Wistar, while NEC exhibit higher levels of anxiety-like behaviour than the control strains.

### Neophobia in the ET

Before conducting the OFT, we assessed the behaviour of the three strains in the ET (Fig. 1c1), a less anxiogenic variation of the OFT used to evaluate neophobia and exploratory behaviour. One-way ANOVA revealed a significant strain effect on the latency to emerge from the central box (F_2,32_=5.05; *p* = 0.012). GAERS exhibited a significantly shorter emergence latency (9.69 ± 1.3s) compared to both Wistar (22.6 ± 4.8; *p* = 0.048) and NEC rats (25.20 ± 3.7; *p* = 0.001) (Fig. 1c2), indicating reduced anxiety-like avoidance behaviour and, therefore, reduced neophobia. Still, the number of entries in the central box did not differ significantly across strains (F_2,32_=0.79; *p* = 0.47) (Fig. 1c3).

In summary, female GAERS display lower anxiety-like behaviour in the ET, compared to Wistar and NEC rats, which show similar levels of anxiety-like behaviour.

### Exploratory Behaviour in the OFT

The OFT (Fig. 1d1) was conducted on day 7 (Fig. 1a) to assess spontaneous locomotor activity and anxiety-like behaviour, complementing the EPM findings (see representative traces in Fig. 1d2). One-way ANOVA revealed no significant strain differences in the number of entries into the central zone (F_2,32_=1.15; *p* = 0.33) (Fig. 1dd3) or in total entries as a measure of exploration (F_2,32_=1.1; *p* = 0.34) (Fig. 1d3).

Analysis of time spent in various zones showed no significant strain effects for the distance covered in the periphery (F_2,32_=0.04; *p* = 0.96), intermediate (F_2,32_=0.06; *p* = 0.94), or central zones (F_2,32_=0.27; *p* = 0.76) (Fig. 1d4).

Regarding exploratory behaviour, a significant strain effect was observed for the distance covered in the periphery (F_2,32_=4.78; *p* = 0.02), with GAERS covering less distance than NEC (1930 ± 190.8 cm vs. 2666 ± 134.2 cm; *p* = 0.01) (Fig. 1d5). No differences were found for distances covered in the intermediate (F_2,32_=0.86; *p* = 0.43), nor central zone (F_2,32_=0.32; *p* = 0.73) (Fig. 1d5). The total distance travelled also differed significantly between strains (F_2,32_=4.45; *p* = 0.02) (Fig. 1d5), with GAERS travelling less than NEC (2476 ± 232.4 cm vs. 3281 ± 139.6; *p* = 0.024) (Fig. 1d5). However, the mean velocity did not differ (Fig. 1d6).

In summary, female GAERS rats show a reduced neophobia profile compared to Wistar and NEC with some evidence of reduced anxiety, as evidenced by the shorter latency to emerge in the ET. No firm conclusion can be drawn for the anxiety-like behaviour of NEC rats since they spent a smaller time in the OA of the EPM (indicative of higher anxiety) but show similar entries in the intermediate and central zones compared to Wistar rats. Regarding locomotion, female GAERS show decrease levels of activity in the periphery and in total exploration, although they do not show reduced velocity and thus normal locomotor behaviour.

### Recognition Memory in the NOR Test

**Fig. 2:**
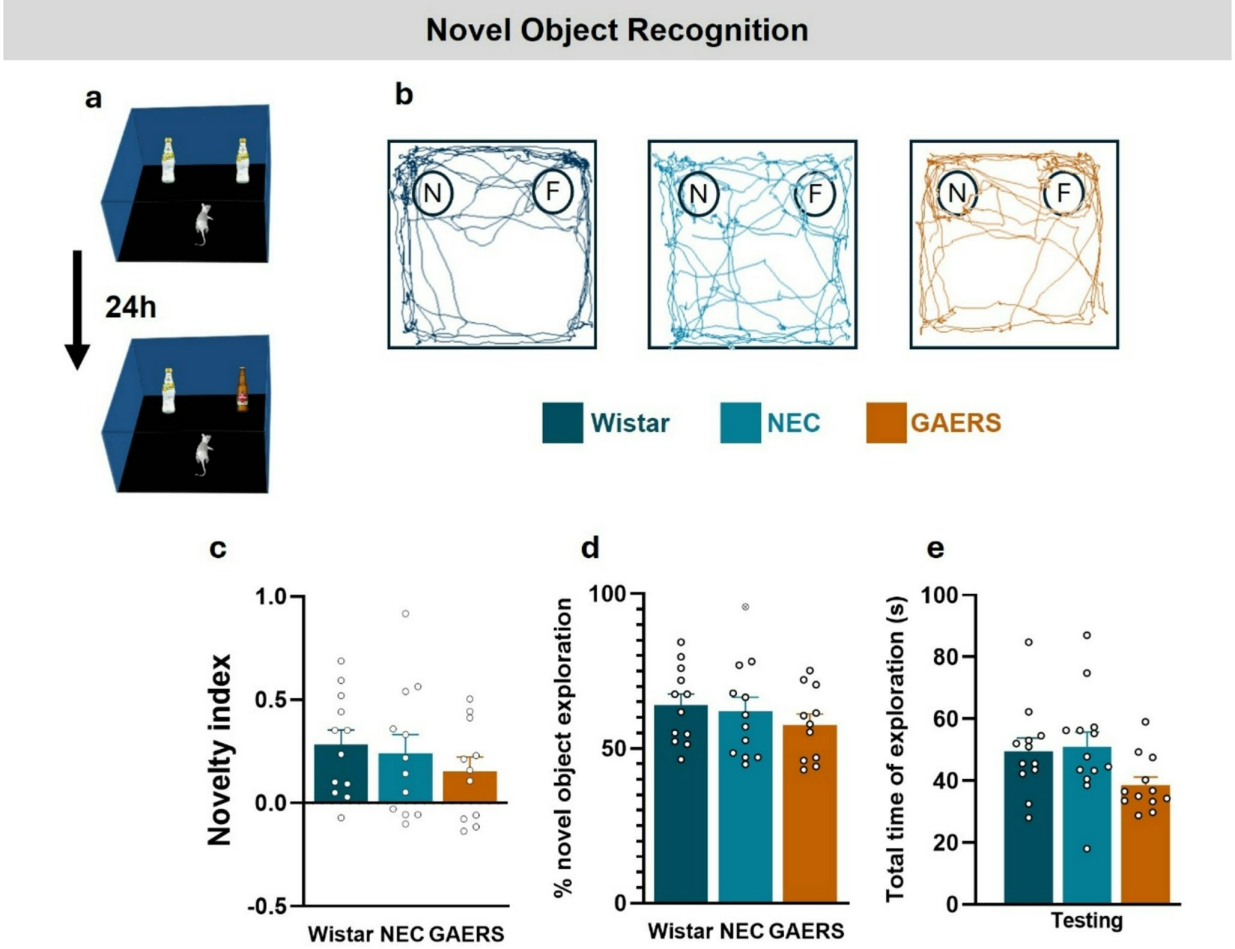
Long-term recognition memory of female GAERS in the NOR test. (**a**) Representation of the arena for the NOR test: rats were presented with a novel object 24 h after the learning session. (**b**) Illustrative traces of the activity of a Wistar, a NEC and GAERS rat during 5 min free exploration. (**c**) Novelty index (see Methods for details). (**d**) Percentage of time spent exploring the novel object. (**e**) Duration of total exploration of the objects (Wistar *n* = 12, NEC *n* = 13, GAERS *n* = 12) (*p < 0.05, **p < 0.001; one-way ANOVA followed by Tukey pairwise multiple comparisons tests)

Following 5 days of handling and 2 days of habituation to the OF arena, the sample trial of the NOR test took place with two objects in the arena (Fig. 1a). Twenty-four hours later the familiar/novel test trial (Fig. 2a) was followed totest long-term recognition memory. In this phase, the total exploration time did not reveal significant strain differences (F_2,31_=2.82; *p* = 0.07), although a non-significant tendency towards a decrease was observed in GAERS compared to NEC (38.65 ± 2.58 vs. 50.98 ± 4.74; *p* = 0.086) (Fig. 2e). Novelty index did not differ between strains (F_2,32_=0.69; *p* = 0.509) (Fig. 2c), with all strains being able to discriminate the novel from the familiar object. This was further confirmed by the percentage of time spent in novel object exploration (F_2,32_=0.687; *p* = 0.510) (Fig. 2d).

In summary, female GAERS rats show similar long-term object recognition memory to NEC and Wistar rats.

### Spatial Working Memory in the Y-Maze

**Fig. 3:**
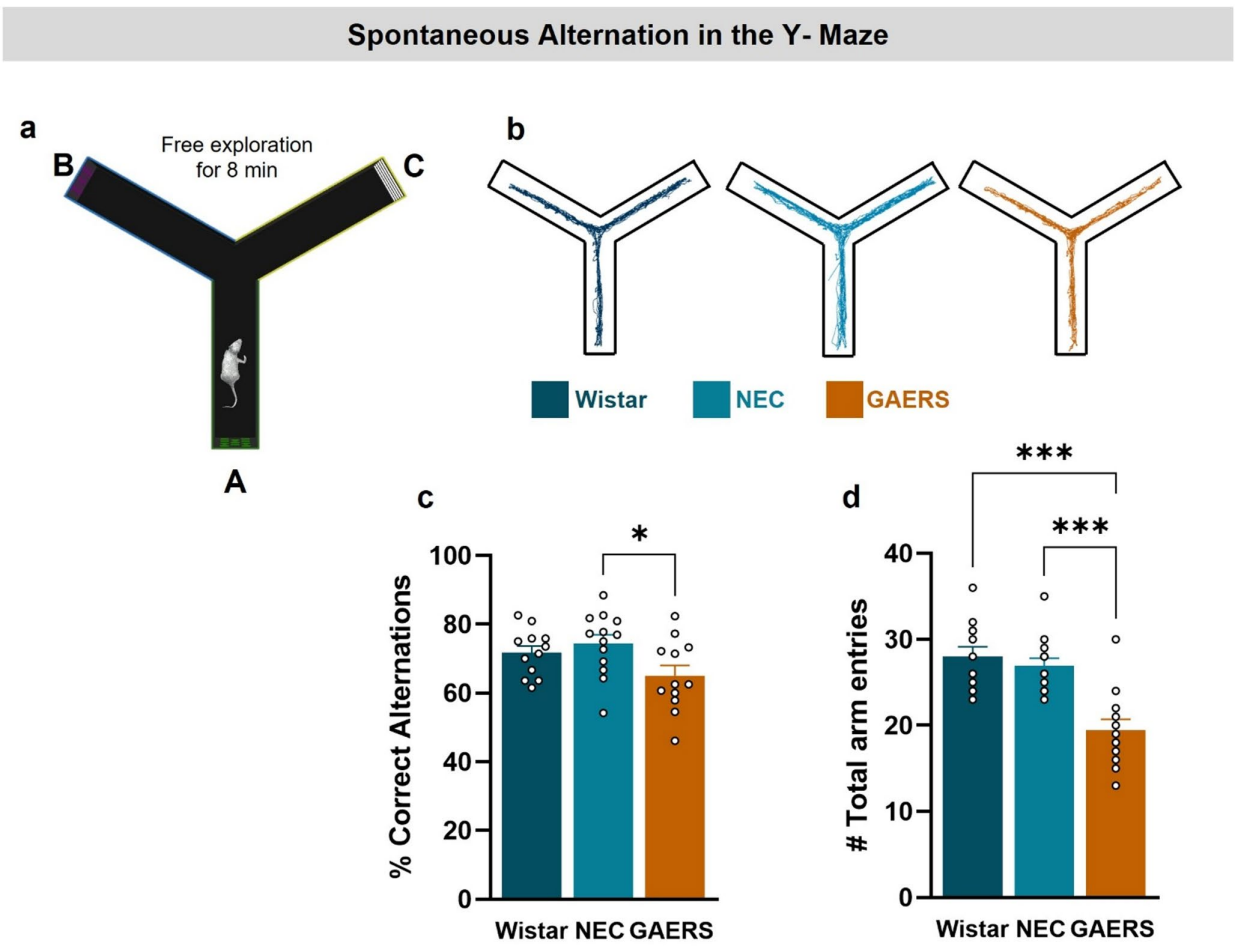
Spatial working memory of female GAERS in the Y-Maze. (**a**) Representation of the Y-Maze and types of alternations during the 8 min free exploration of the Y-Maze. (**b**) Illustrative traces of the activity of a Wistar, a NEC and GAERS rats during 5 min free exploration. (**c**) Number of correct alternations (percentage). (**d**) Total number of entries (Wistar *n* = 12, NEC *n* = 13, GAERS *n* = 12) (*p < 0.05, **p < 0.01, ***p < 0.001, one-way ANOVA followed by Tukey pairwise multiple comparisons tests)

An index of spatial working memory was acquired as spontaneous alternations in the Y-Maze (Fig. 1a). Performance was above chance level (i.e., >50%) in all strains. Nevertheless, one-way ANOVA revealed significant differences between strains (F_2,34_=3.63; *p* = 0.037), with post-hoc comparisons showing a significant decrease in correct alternations for GAERS compared to NEC (65.08 ± 2.99% vs. 74.45 ± 2.5%; *p* = 0.033) (Fig. 3c). Furthermore, one-way ANOVA detected a difference in total arm entries between strains (F _2,34_=16.98; *p* < 0.001) GAERS show lower exploration compared to Wistar (19.42 ± 1.31 vs. 28 ± 1.16; *p* < 0.001) and NEC (26.92 ± 0.91; *p* < 0.001) (Fig. 3d), confirming the reduction of exploratory behaviour in the epileptic strain.

In summary, female GAERS rats showed a reduction in correct spontaneous alternations compared to NEC and total exploration compared to NEC and Wistar females, a finding consistent with impaired working memory.

## Discussion

**Fig. 4:**
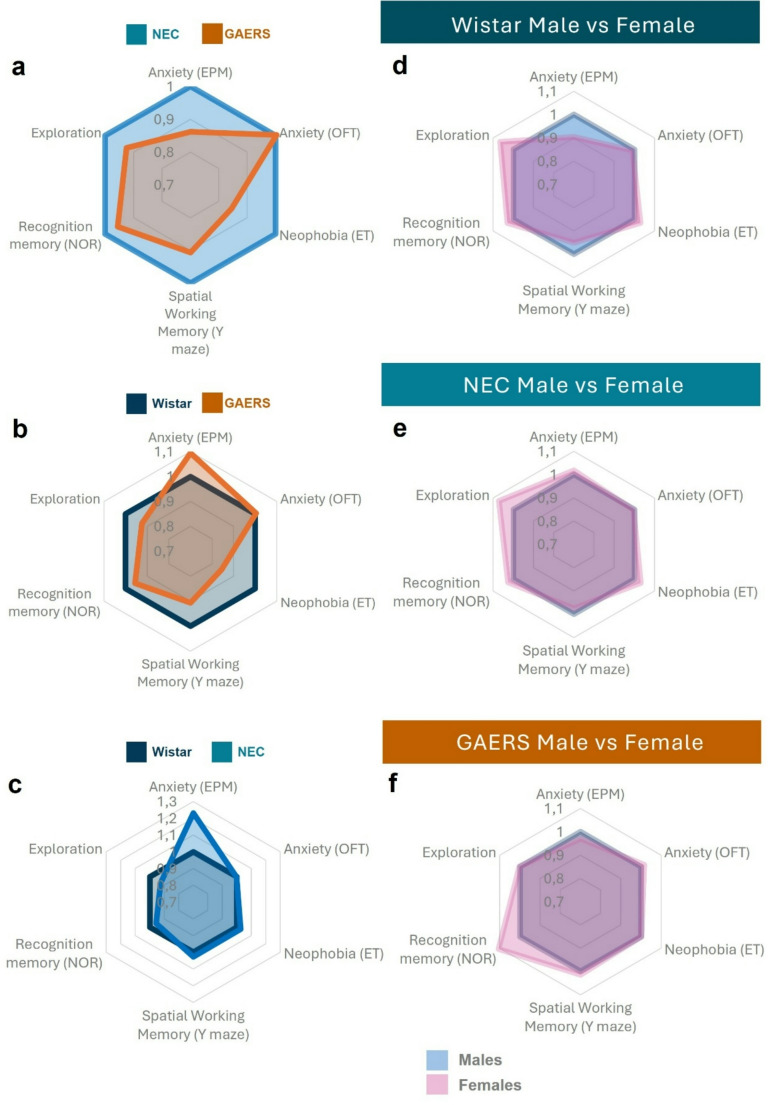
Schematic summary of anxiety and memory in female GAERS, NEC and Wistar rats and comparison with those of male rats of the same strains. **a**-**c**) Schematic radial plots comparing the results of female GAERS, NEC and Wistar rats. Data are normalized to NEC (**a**) and Wistar (**b**, **c**). Each point corresponds to either an anxiety or a memory test, as illustrated. Data points < 1 and > 1 indicate a lower and a higher execution, respectively. **d**-**e**) Schematic radial plots comparing the results of male and female rats of the Wistar (**d**), NEC (**e**), and GAERS (**f**) strains (data of male GAERS, NEC and Wistar rats are from Neuparth-Sottomayor et al., 2023). Data are normalized to males Wistar (**d**), males NEC (**e**), and males GAERS (**f**). (**a**) Compared to female NEC, female GAERS show lower anxiety-like behaviour in 2 out of 3 anxiety tests, reduced exploration levels and are deficient in spatial working memory but not in recognition memory. (**b**) Compared to female Wistar, female GAERS show low levels of anxiety and neophobia, reduced exploration levels. (**c**) Compared to female Wistar, female NEC show high anxiety-like behaviour in one out of 3 tests and show similar levels of cognitive performance. (**d**) Compared to males, female Wistar show reduced anxiety-like behaviour and increased levels of exploration in the Y maze. (**e**) Compared to males, female NEC show higher level of exploration in the OFT. (**f**) Compared to males, female GAERS show lower levels of anxiety-like behaviour and notably increased recognition memory

The main findings of this study (Fig. 4a-c) are that female GAERS rats (1) display normal anxiety-like behaviours relative to two non-epileptic control strains with some evidence of reduced neophobia, (2) show deficits in a spatial working memory task, but (3) exhibit long-term recognition memory similar to the control strains. This behavioural profile differs from that of male GAERS rats from the same Cardiff colony [31] (Fig. 4d-f), which demonstrated lower levels of anxiety-like behaviour and neophobia and impairments in both recognition and spatial working memory. To our knowledge, this is the first systematic investigation of anxiety and cognition in the female GAERS rats, thus providing important information concerning sex-related changes in cognition in children with CAE.

### Comorbid Anxiety

The present study found normal, and in some cases reduced, anxiety-like behaviour in GAERS females compared to NEC rats, particularly in the EPM and ET. This suggests that the spatial alternation deficits in female GAERS rats are unlikely to be confounded by heightened anxiety. These findings align with evidence of normal/lower anxiety-like behaviour in male GAERS compared to NEC and outbred Wistar rats [31, 34]. Male and female GAERS from the Australia colony have high levels of anxiety and depressive-like behaviour compared to controls [29, 30, 42–44], whereas in the Canadian colony the results are more mixed with some studies reporting normal or increased anxiety-like behaviour in female GAERS [22, 23, 27, 28, 45]. Although housing conditions—such as temperature, humidity, and the duration of the light-dark cycle were similar across all studies, the differences in behavioural outcomes may also reflect colony-specific factors, including genetic drift or subtle variations in breeding practices [46].

### Recognition Memory: a Sex-specific Difference in Rats with Spontaneous ASs

Our finding that female GAERS rats show unimpaired long-term recognition memory contrasts with the results in male GAERS (Fig. 4) in a previous work [31]. Sex differences in learning have been noted in other tasks. For example, previous studies have shown that male GAERS have impairments in reversal learning and visual discrimination tasks and learn these tasks more slowly and make more errors than female GAERS and NEC rats [27]. The pattern of spared object recognition in female GAERS rats suggests that the function of perirhinal cortex may be relatively preserved even in the presence of ASs. This may reflect altered levels of hormones, glutamate receptors and GABAergic inhibition, and/or alterations in intracellular signalling pathway that are known to modulate synaptic plasticity [47–50].

Research on sex differences in cognitive function is a key factor in ASs studies, particularly for models that exhibit SWDs. Research in other rat strains with SWDs, such as Hooded Lister and Fisher 344 rats, provide insights into potential sex-specific patterns that may apply to GAERS. Notably, female hooded Lister rats outperform males in non-spatial working memory tasks, while their spatial performance varies with the estrous cycle, peaking when estrogen and progesterone levels are low [51]. Similarly, middle-aged female Fisher 344 rats show more robust spatial long-term memory than males, specifically under chronic stress, possibly due to estrogen’s protective effects [52–54]. However, the consistent presence of SWDs in these strains may modulate cognition, suggesting that sex differences in GAERS could similarly reflect interactions between hormonal factors and disruption caused by epileptiform activity.

### Potential Mechanisms of Female GAERS Working Memory Impairment and Sex-specific Differences

The most striking result in our experiment is the dissociation between working memory and recognition memory in female GAERS rats. Deficits in working memory in female GAERS were detected in the spontaneous alternation task in the Y-Maze in the current study, and also in the male GAERS from our laboratory [31] (Fig. 4). Moreover, female GAERS showed lower arm exploration compared to female Wistar and NEC rats, confirming a reduced exploratory behaviour in the epileptic strain. Our previous work highlighted pathophysiological mechanisms contributing to memory deficits in GAERS, but sex-specific differences, particularly in working and recognition memory warrant further exploration.

GAERS exhibit genetic, cellular, and neuronal network abnormalities compared to NEC and Wistar rats (see recent reviews by [55–57]). These abnormalities disrupt theta and gamma oscillations, which are essential for working memory, by interfering with sensory and cognitive integration in the hippocampus and prefrontal cortex [58, 59]. However, the absence of recognition memory deficits in female GAERS point to sex-specific factors that may protect perirhinal cortex function, a key region for object recognition [60]. Recognition memory relies on long-term depression (LTD) in the perirhinal cortex [61, 62]. In male GAERS, frequent SWDs may impair LTD by altering glutamatergic and GABAergic signalling, leading to recognition memory deficits [63]. In contrast, female GAERS may be protected by sex hormones, particularly estrogen, which modulates synaptic plasticity. Estrogen enhances hippocampal and cortical plasticity, potentially counteracting seizure-induced LTD impairments in the perirhinal cortex [64–66]. For example, low estrogen levels during estrous phases improve discrimination in fear-conditioning tasks, aligning performance with males [67], while higher progesterone levels during behavioural estrus or pregnancy improve object recognition and placement tasks [68]. Although speculative, these hormonal effects may stabilize LTD in female GAERS, preserving recognition memory despite SWDs. Moreover, neurosteroids, particularly allopregnalone and androstanediol may lead to sex-specific alterations in GABAergic mechanisms, by modulating GABAergic inhibition and mitigating seizure-induced excitability in the perirhinal cortex, further protecting recognition memory [20, 69]. Additionally, activation of progesterone and oestrogen receptors increase GABA_A_ receptor-mediated tonic current in males but not female mice [70].

These findings suggest that sex-specific differences in GAERS memory performance may arise from an interplay between AS-induced network dysfunction and hormonal modulation of synaptic plasticity. Future studies should investigate how estrogen and neurosteroids influence perirhinal cortex LTD in GAERS to confirm these mechanisms and explore therapeutic targets for sex-specific cognitive comorbidities in ASs.

## Conclusions

In summary, female GAERS rats exhibit anxiety-like behaviour comparable to males and selective vulnerability to working memory, while males show a more widespread cognitive impairment. Further research should explore the mechanisms underlying the selective vulnerability in working memory and spared recognition memory in female GAERS rats, such as the role of sex hormones, GABAergic inhibition, prefrontal cortical and hippocampal networks. Addressing these crucial questions will help overcome our current knowledge gap regarding the interactions between sex, epilepsy and associated cognitive comorbidities.

Taken together, our findings align with accumulating evidence of neuropsychological comorbidities in children with CAE, including deficits in attention, executive function, such as cognitive flexibility and planning [11–14, 71]. In both children with CAE and animal models of ASs males and females show poor working memory, indicating a strong parallel in this cognitive domain. Recognition memory, however, remains relatively intact in children with CAE, suggesting resilience in certain cognitive domains despite impairments in other areas [11, 35]. In GAERS rats, recognition memory deficits are observed, particularly in tasks requiring integration of sensory information, such as crossmodal object recognition in male GAERS rats [25, 31]. In contrast, female GAERS show intact recognition memory, which is similar to the CAE phenotype, although in future they should be tested in crossmodal object recognition task to evaluate this further. This parallel suggests that certain cognitive domains may be more resilient to seizure-related disruptions across species and, the parallels in working memory deficits highlight the predictive validity of these models in studying comorbidities in both males and females with ASs. Investigating sex/gender differences in human and experimental ASs will enhance our understanding of their neuropsychological comorbidities and support the development of patient-tailored therapies aimed at alleviating both seizures and cognitive impairments.

## Data Availability

No datasets were generated or analysed during the current study.

## References

[1] Carlson C, Dugan P, Kirsch HE, Friedman D (2014) Sex differences in seizure types and symptoms. Epilepsy Behav 41:103–108. 10.1016/j.yebeh.2014.09.051 25461198 10.1016/j.yebeh.2014.09.051 PMC4267158

[2] Asadi-Pooya AA, Alkhaldi M, Damabi NM, Dehkordi KF (2024) Sex differences in epilepsies: a narrative review. Archives Epilepsy 30:100–103. 10.4274/ARCHEPILEPSY.2024.24124

[3] McHugh JC, Delanty N (2008) Chap. 2 epidemiology and classification of epilepsy. Gender comparisons. Int Rev Neurobiol 83:11–26. 10.1016/S0074-7742(08)00002-0 18929074 10.1016/S0074-7742(08)00002-0

[4] Christensen J, Kjeldsen MJ, Andersen H, Friis ML, Sidenius P (2005) Gender differences in epilepsy. Epilepsia 46:956–960. 10.1111/J.1528-1167.2005.51204.X 15946339 10.1111/j.1528-1167.2005.51204.x

[5] Zhong R, Chen Q, Li M, Li N, Zhang X, Lin W (2021) Sex differences in anxiety in patients with epilepsy: status and risk factors analysis. Epilepsy Behav 116. 10.1016/j.yebeh.2021.107801 10.1016/j.yebeh.2021.107801 33578225

[6] Liu Z, Yin R, Fan Z, Fan H, Wu H, Shen B, Wu S, Kuang F (2020) Gender differences in associated and predictive factors of anxiety and depression in people with epilepsy. Front Psychiatry 11. 10.3389/FPSYT.2020.00670 10.3389/fpsyt.2020.00670 PMC7365887 32754069

[7] Gaus V, Kiep H, Holtkamp M, Burkert S, Kendel F (2015) Gender differences in depression, but not in anxiety in people with epilepsy. Seizure 32:37–42. 10.1016/j.seizure.2015.07.012 26552559 10.1016/j.seizure.2015.07.012

[8] Chai X, Xiao Z, Zhao Q, Wang J, Ding D, Zhang J (2023) Cognitive impairment as a comorbidity of epilepsy in older adults: analysis of global and domain-specific cognition. Epileptic Disord 25:65–73. 10.1002/EPD2.20057 37013261 10.1002/epd2.20057

[9] Crunelli V, Lorincz ML, McCafferty C, Lambert RC, Leresche N, Di Giovanni G, David F (2020) Clinical and experimental insight into pathophysiology, comorbidity and therapy of absence seizures. Brain 143:2341–2368. 10.1093/brain/awaa072 32437558 10.1093/brain/awaa072 PMC7447525

[10] Crunelli V, Leresche N (2002) Childhood absence epilepsy: genes, channels, neurons and networks. Nat Rev Neurosci 3:371–382. 10.1038/nrn811 11988776 10.1038/nrn811

[11] Caplan R, Siddarth P, Stahl L, Lanphier E, Vona P, Gurbani S, Koh S, Sankar R, Shields WD (2008) Childhood absence epilepsy: behavioral, cognitive, and linguistic comorbidities. Epilepsia 49:1838–1846. 10.1111/J.1528-1167.2008.01680.X 18557780 10.1111/j.1528-1167.2008.01680.x

[12] D’Agati E, Cerminara C, Casarelli L, Pitzianti M, Curatolo P (2012) Attention and executive functions profile in childhood absence epilepsy. Brain Dev 34:812–817. 10.1016/J.BRAINDEV.2012.03.001 22459253 10.1016/j.braindev.2012.03.001

[13] Jones JE, Watson R, Sheth R, Caplan R, Koehn M, Seidenberg M, Hermann B (2007) Psychiatric comorbidity in children with new onset epilepsy. Dev Med Child Neurol 49:493–497. 10.1111/J.1469-8749.2007.00493.X 17593119 10.1111/j.1469-8749.2007.00493.x

[14] Masur D, Shinnar S, Cnaan A, Shinnar RC, Clark P, Wang J, Weiss EF, Hirtz DG, Glauser TA (2013) Pretreatment cognitive deficits and treatment effects on attention in childhood absence epilepsy. Neurology 81:1572–1580. 10.1212/WNL.0B013E3182A9F3CA 24089388 10.1212/WNL.0b013e3182a9f3ca PMC3806916

[15] Lorkiewicz SA, Modiano YA, Miller BI, Van Cott AC, Haneef Z, Sullivan-Baca E (2024) The neuropsychological presentation of women with epilepsy: clinical considerations and future directions. Clin Neuropsychologist 38:1382–1408. 10.1080/13854046.2023.2283937 10.1080/13854046.2023.2283937 37993977

[16] Berger J, Oltmanns F, Holtkamp M, Bengner T (2017) Sex differences in verbal and nonverbal learning before and after Temporal lobe epilepsy surgery. Epilepsy Behav 66:57–63. 10.1016/j.yebeh.2016.11.037 28033547 10.1016/j.yebeh.2016.11.037

[17] Bengner T, Fortmeier C, Malina T, Lindenau M, Voges B, Goebell E, Stodieck S (2006) Sex differences in face recognition memory in patients with temporal lobe epilepsy, patients with generalized epilepsy, and healthy controls. Epilepsy Behav 9:593–600. 10.1016/j.yebeh.2006.08.021 17088107 10.1016/j.yebeh.2006.08.021

[18] Baxendale S, Heaney D, Thompson PJ, Duncan JS (2010) Cognitive consequences of childhood-onset temporal lobe epilepsy across the adult lifespan. Neurology 75:705–711. 10.1212/WNL.0B013E3181EEE3F0 20733146 10.1212/WNL.0b013e3181eee3f0

[19] Marescaux C, Vergnes M, Depaulis A (1992) Genetic absence epilepsy in rats from Strasbourg - a review. J Neural Transmission Supplement 35:37–69. 10.1007/978-3-7091-9206-1_4 10.1007/978-3-7091-9206-1_4 1512594

[20] Van Luijtelaar G, Yilmaz Onat F, Gallagher MJ (2014) Animal models of absence epilepsies: what do they model and do sex and sex hormones matter? Neurobiol Dis 72:167–179. 10.1016/j.nbd.2014.08.014 25132554 10.1016/j.nbd.2014.08.014 PMC4252718

[21] Roebuck AJ, Greba Q, Onofrychuk TJ, McElroy DL, Sandini TM, Zagzoog A, Simone J, Cain SM, Snutch TP, Laprairie RB, Howland JG (2022) Dissociable changes in spike and wave discharges following exposure to injected cannabinoids and smoked cannabis in genetic absence epilepsy rats from Strasbourg. Eur J Neurosci 55:1063–1078. 10.1111/EJN.15096 33370468 10.1111/ejn.15096

[22] Marks WN, Cain SM, Snutch TP, Howland JG (2016) The T-type calcium channel antagonist Z944 rescues impairments in crossmodal and visual recognition memory in genetic absence epilepsy rats from Strasbourg. Neurobiol Dis 94:106–115. 10.1016/j.nbd.2016.06.001 27282256 10.1016/j.nbd.2016.06.001

[23] Henbid MT, Marks WN, Collins MJ, Cain SM, Snutch TP, Howland JG (2017) Sociability impairments in genetic absence epilepsy rats from strasbourg: reversal by the T-type calcium channel antagonist Z944. Exp Neurol 296:16–22. 10.1016/J.EXPNEUROL.2017.06.022 28658605 10.1016/j.expneurol.2017.06.022

[24] Marques-Carneiro JE, Faure JB, Cosquer B, Koning E, Ferrandon A, De Vasconcelos AP, Cassel JC, Nehlig A (2014) Anxiety and locomotion in genetic absence epilepsy rats from Strasbourg (GAERS): inclusion of Wistar rats as a second control. Epilepsia 55:1460–1468. 10.1111/EPI.12738 25059093 10.1111/epi.12738

[25] Studer F, Laghouati E, Jarre G, David O, Pouyatos B, Depaulis A (2019) Sensory coding is impaired in rat absence epilepsy. J Physiol 597:951–966. 10.1113/JP277297 30548850 10.1113/JP277297 PMC6355637

[26] Getova D, Bowery NG, Spassov V (1997) Effects of GABA(B) receptor antagonists on learning and memory retention in a rat model of absence epilepsy. Eur J Pharmacol 320:9–13. 10.1016/S0014-2999(96)00877-1 9049596 10.1016/s0014-2999(96)00877-1

[27] Roebuck AJ, An L, Marks WN, Sun N, Snutch TP, Howland JG (2020) Cognitive impairments in touchscreen-based visual discrimination and reversal learning in genetic absence epilepsy rats from Strasbourg. Neuroscience 430:105–112. 10.1016/j.neuroscience.2020.01.028 32017953 10.1016/j.neuroscience.2020.01.028

[28] Marks WN, Cavanagh ME, Greba Q, Cain SM, Snutch TP, Howland JG (2016) The genetic absence epilepsy rats from Strasbourg model of absence epilepsy exhibits alterations in fear conditioning and latent inhibition consistent with psychiatric comorbidities in humans. Eur J Neurosci 43:25–40. 10.1111/ejn.13110 26490879 10.1111/ejn.13110

[29] Jones NC, Salzberg MR, Kumar G, Couper A, Morris MJ, O’Brien TJ (2008) Elevated anxiety and depressive-like behavior in a rat model of genetic generalized epilepsy suggesting common causation. Exp Neurol 209:254–260. 10.1016/J.EXPNEUROL.2007.09.026 18022621 10.1016/j.expneurol.2007.09.026

[30] Jones NC, Martin S, Megatia I, Hakami T, Salzberg MR, Pinault D, Morris MJ, O’Brien TJ, van den Buuse M (2010) A genetic epilepsy rat model displays endophenotypes of psychosis. Neurobiol Dis 39:116–125. 10.1016/j.nbd.2010.02.001 20153428 10.1016/j.nbd.2010.02.001

[31] Neuparth-Sottomayor M, Pina CC, Morais TP, Farinha-Ferreira M, Abreu DS, Solano F, Mouro F, Good M, Sebastião AM, Di Giovanni G, Crunelli V, Vaz SH (2023) Cognitive comorbidities of experimental absence seizures are independent of anxiety. Neurobiol Dis 186. 10.1016/J.NBD.2023.106275 10.1016/j.nbd.2023.106275 37648038

[32] Cassar D, Radic M, Casarrubea M, Crunelli V, Di Giovanni G (2022) The effect of cannabinoid receptor agonist WIN 55,212-2 on anxiety-like behavior and locomotion in a genetic model of absence seizures in the elevated plus-maze. CNS Neurosci Ther 28:1268–1270. 10.1111/CNS.13848 35470960 10.1111/cns.13848 PMC9253729

[33] De Deurwaerdère P, Casarrubea M, Cassar D, Radic M, Puginier E, Chagraoui A, Crescimanno G, Crunelli V, Di Giovanni G (2022) Cannabinoid 1/2 receptor activation induces strain-dependent behavioral and neurochemical changes in genetic absence epilepsy rats from strasbourg and non-epileptic control rats. Front Cell Neurosci 16. 10.3389/FNCEL.2022.886033 10.3389/fncel.2022.886033 PMC9169225 35677756

[34] Casarrubea M, Radic M, Morais TP, Mifsud E, Cuboni E, Aiello S, Crescimanno G, Crunelli V, Di Giovanni G (2024) A quantitative and T-pattern analysis of anxiety-like behavior in male GAERS, NEC, and Wistar rats bred under the same conditions, against a commercially available Wistar control group in the hole board and elevated plus maze tests. CNS Neurosci Ther 30. 10.1111/CNS.14443 10.1111/cns.14443 PMC10916429 37658671

[35] Sitnikova E (2024) Behavioral and cognitive comorbidities in genetic rat models of absence epilepsy (Focusing on GAERS and wag/rij rats). Biomedicines 12. 10.3390/BIOMEDICINES12010122 10.3390/biomedicines12010122 PMC10812980 38255227

[36] Sarkisova K, van Luijtelaar G (2011) The wag/rij strain: a genetic animal model of absence epilepsy with comorbidity of depression [corrected]. Prog Neuropsychopharmacol Biol Psychiatry 35:854–876. 10.1016/J.PNPBP.2010.11.010 21093520 10.1016/j.pnpbp.2010.11.010

[37] Brock JW, Bond SP, Ross KD, Farooqui SM, Kloster CA (1996) Abnormal behaviors in the stargazer rat are maladaptive, but not anxiety related. Physiol Behav 59:1011–1014. 10.1016/0031-9384(95)02170-1 8778837 10.1016/0031-9384(95)02170-1

[38] Behavioral characteristics of female Wag/Rij rats - PubMed, https://pubmed.ncbi.nlm.nih.gov/22145338/22145338

[39] Pellow S, Chopin P, File SE, Briley M (1985) Validation of open:closed arm entries in an elevated plus-maze as a measure of anxiety in the rat. J Neurosci Methods 14:149–167. 10.1016/0165-0270(85)90031-7 2864480 10.1016/0165-0270(85)90031-7

[40] Honig WK (2018) Studies of working memory in the pigeon. Cognitive processes in animal behavior. 211–248. 10.4324/9780203710029-8/STUDIES-WORKING-MEMORY-PIGEON-WERNER-HONIG

[41] Honig Wk (1978) Studies of working memory in the pigeon. In: Fowler HSH, Honig H (eds) Cognitive processes in animal cognition. Lawrence Erlbaum Associates, Inc., K. (New Jersey, NJ, pp 211–248

[42] Dezsi G, Ozturk E, Stanic D, Powell KL, Blumenfeld H, O’Brien TJ, Jones NC (2013) Ethosuximide reduces epileptogenesis and behavioral comorbidity in the GAERS model of genetic generalized epilepsy. Epilepsia 54:635–643. 10.1111/EPI.12118 23464801 10.1111/epi.12118 PMC3618492

[43] Bouilleret V, Hogan RE, Velakoulis D, Salzberg MR, Wang L, Egan GF, O’Brien TJ, Jones NC (2009) Morphometric abnormalities and hyperanxiety in genetically epileptic rats: a model of psychiatric comorbidity? Neuroimage. 45:267–274. 10.1016/J.NEUROIMAGE.2008.12.019 10.1016/j.neuroimage.2008.12.019 19167503

[44] Powell KL, Tang H, Ng C, Guillemain I, Dieuset G, Dezsi G, Çarçak N, Onat F, Martin B, O’Brien TJ, Depaulis A, Jones NC (2014) Seizure expression, behavior, and brain morphology differences in colonies of genetic absence epilepsy rats from Strasbourg. Epilepsia 55:1959–1968. 10.1111/EPI.12840 25377760 10.1111/epi.12840

[45] Marks WN, Zabder NK, Greba Q, Cain SM, Snutch TP, Howland JG (2019) The T-type calcium channel blocker Z944 reduces conditioned fear in genetic absence epilepsy rats from Strasbourg and the non-epileptic control strain. Eur J Neurosci 50:3046–3059. 10.1111/EJN.14406 30889299 10.1111/ejn.14406

[46] Dezsi G, Ozturk E, Salzberg MR, Morris M, O’Brien TJ, Jones NC (2016) Environmental enrichment imparts disease-modifying and transgenerational effects on genetically-determined epilepsy and anxiety. Neurobiol Dis 93:129–136. 10.1016/j.nbd.2016.05.005 27185593 10.1016/j.nbd.2016.05.005

[47] Albasser MM, Olarte-Sánchez CM, Amin E, Brown MW, Kinnavane L, Aggleton JP (2015) Perirhinal cortex lesions in rats: novelty detection and sensitivity to interference. Behav Neurosci 129:227–243. 10.1037/BNE0000049 26030425 10.1037/bne0000049 PMC4450885

[48] Biagini G, D’Antuono M, Benini R, de Guzman P, Longo D, Avoli M (2013) Perirhinal cortex and temporal lobe epilepsy. Front Cell Neurosci 7. 10.3389/FNCEL.2013.00130 10.3389/fncel.2013.00130 PMC3756799 24009554

[49] Luine V, Mohan G, Attalla S, Jacome L, Frankfurt M (2022) Androgens enhance recognition memory and dendritic spine density in the Hippocampus and prefrontal cortex of ovariectomized female rats. Neuroscience 568. 10.1016/j.neuroscience.2022.06.002 10.1016/j.neuroscience.2022.06.002 PMC9719572 35671881

[50] Avoli M, de Curtis M, Lévesque M, Librizzi L, Uva L, Wang S (2022) GABAA signaling, focal epileptiform synchronization and epileptogenesis. Front Neural Circuits 16. 10.3389/FNCIR.2022.984802 10.3389/fncir.2022.984802 PMC9581276 36275847

[51] Sutcliffe JS, Marshall KM, Neill JC (2007) Influence of gender on working and spatial memory in the novel object recognition task in the rat. Behav Brain Res 177:117–125. 10.1016/j.bbr.2006.10.029 17123641 10.1016/j.bbr.2006.10.029

[52] Colettis NC, Habif M, Oberholzer MV, Filippin F, Jerusalinsky DA (2022) Differences in learning and memory between middle-aged female and male rats. Learn Memory 29:120–125. 10.1101/LM.053578.122 10.1101/lm.053578.122 PMC9053109 35428728

[53] Luine V (2002) Sex differences in chronic stress effects on memory in rats. Stress 5:205–216. 10.1080/1025389021000010549 12186683 10.1080/1025389021000010549

[54] Luine V, Gomez J, Beck K, Bowman R (2017) Sex differences in chronic stress effects on cognition in rodents. Pharmacol Biochem Behav 152:13–19. 10.1016/j.pbb.2016.08.005 27566290 10.1016/j.pbb.2016.08.005 PMC5195878

[55] Lindquist BE, Timbie C, Voskobiynyk Y, Paz JT (2023) Thalamocortical circuits in generalized epilepsy: pathophysiologic mechanisms and therapeutic targets. Neurobiol Dis 181:106094. 10.1016/j.nbd.2023.106094 36990364 10.1016/j.nbd.2023.106094 PMC10192143

[56] Crunelli V, Lőrincz ML, McCafferty C, Lambert RC, Leresche N, Di Giovanni G, David F (2020) Clinical and experimental insight into pathophysiology, comorbidity and therapy of absence seizures. Brain. 10.1093/brain/awaa072 32437558 10.1093/brain/awaa072 PMC7447525

[57] Crunelli V, David F, Morais TP, Lorincz ML (2023) HCN channels and absence seizures. Neurobiol Dis 181:106107. 10.1016/j.nbd.2023.106107 37001612 10.1016/j.nbd.2023.106107

[58] Arski ON, Young JM, Smith M, Lou, Ibrahim GM (2021) The oscillatory basis of working memory function and dysfunction in epilepsy. Front Hum Neurosci 14. 10.3389/FNHUM.2020.612024 10.3389/fnhum.2020.612024 PMC7874181 33584224

[59] Cao F, Liu JJ, Zhou S, Cortez MA, Snead OC, Han J, Jia Z (2020) Neuroligin 2 regulates absence seizures and behavioral arrests through GABAergic transmission within the thalamocortical circuitry. Nat Commun 11. 10.1038/S41467-020-17560-3 10.1038/s41467-020-17560-3 PMC7385104 32719346

[60] Warburton EC, Brown MW (2015) Neural circuitry for rat recognition memory. Behav Brain Res 285:131–139. 10.1016/j.bbr.2014.09.050 25315129 10.1016/j.bbr.2014.09.050 PMC4383363

[61] Warburton EC, Koder T, Cho K, Massey PV, Duguid G, Barker GRI, Aggleton JP, Bashir ZI, Brown MW (2003) Cholinergic neurotransmission is essential for perirhinal cortical plasticity and recognition memory. Neuron 38:987–996. 10.1016/S0896-6273(03)00358-1 12818183 10.1016/s0896-6273(03)00358-1

[62] Griffiths S, Scott H, Glover C, Bienemann A, Ghorbel MT, Uney J, Brown MW, Warburton EC, Bashir ZI (2008) Expression of long-term depression underlies visual recognition memory. Neuron 58:186–194. 10.1016/j.neuron.2008.02.022 18439404 10.1016/j.neuron.2008.02.022

[63] Lindquist BE, Timbie C, Voskobiynyk Y, Paz JT (2023) Thalamocortical circuits in generalized epilepsy: pathophysiologic mechanisms and therapeutic targets. Neurobiol Dis 181. 10.1016/j.nbd.2023.106094 10.1016/j.nbd.2023.106094 PMC10192143 36990364

[64] Baquedano LE, Bernal EG, Carrion DJ, Delgado AD, Gavidia CM, Kirwan DE, Gilman RH, Verastegui MR (2023) Impaired Spatial working memory and reduced hippocampal neuronal density in a rat model of neurocysticercosis. Front Cell Neurosci 17. 10.3389/FNCEL.2023.1183322 10.3389/fncel.2023.1183322 PMC10267319 37323586

[65] Llorente R, Marraudino M, Carrillo B, Bonaldo B, Simon-Areces J, Abellanas-Pérez P, Rivero-Aguilar M, Fernandez-Garcia JM, Pinos H, Garcia-Segura LM, Collado P, Grassi D (2020) G Protein-Coupled Estrogen receptor immunoreactivity fluctuates during the estrous cycle and show sex differences in the amygdala and dorsal Hippocampus. Front Endocrinol (Lausanne) 11. 10.3389/FENDO.2020.00537 10.3389/fendo.2020.00537 PMC7426398 32849310

[66] Tada H, Koide M, Ara W, Shibata Y, Funabashi T, Suyama K, Goto T, Takahashi T (2015) Estrous cycle-dependent phasic changes in the stoichiometry of hippocampal synaptic AMPA receptors in rats. PLoS ONE 10. 10.1371/JOURNAL.PONE.0131359 10.1371/journal.pone.0131359 PMC4486186 26121335

[67] Trask S, Reis DS, Ferrara NC, Helmstetter FJ (2020) Decreased cued fear discrimination learning in female rats as a function of estrous phase. Learn Memory 27:254–257. 10.1101/LM.051185.119 10.1101/lm.051185.119 PMC7233151 32414943

[68] Paris JJ, Frye CA (2008) Estrous cycle, pregnancy, and parity enhance performance of rats in object recognition or object placement tasks. Reproduction 136:105–115. 10.1530/REP-07-0512 18390689 10.1530/REP-07-0512 PMC2556072

[69] Reddy DS (2017) The neuroendocrine basis of sex differences in epilepsy. Pharmacol Biochem Behav 152:97–104. 10.1016/j.pbb.2016.07.002 27424276 10.1016/j.pbb.2016.07.002

[70] Vázquez-Sola A, Torres-Torrelo H, Yagüe JG (2025) Membrane progesterone and oestrogen receptors modulate GABAergic transmission in the prefrontal cortex of prepubertal male, but not female, mice. Exp Physiol. 10.1113/EP092439 40309895 10.1113/EP092439 PMC12128468

[71] Fonseca Wald ELA, Hendriksen JGM, Drenthen GS, Kuijk SMJV, Aldenkamp AP, Vles JSH, Vermeulen RJ, Debeij-van Hall MHJA, Klinkenberg S (2019) Towards a better understanding of cognitive deficits in absence epilepsy: a systematic review and meta-analysis. Neuropsychol Rev 29:421–449. 10.1007/S11065-019-09419-2 31776780 10.1007/s11065-019-09419-2 PMC6892766

